# The role of survival motor neuron protein (SMN) in protein homeostasis

**DOI:** 10.1007/s00018-018-2849-1

**Published:** 2018-06-05

**Authors:** Helena Chaytow, Yu-Ting Huang, Thomas H. Gillingwater, Kiterie M. E. Faller

**Affiliations:** 1 Euan MacDonald Centre for Motor Neurone Disease Research, University of Edinburgh, Edinburgh, UK; 2 Edinburgh Medical School: Biomedical Sciences, University of Edinburgh, Edinburgh, UK; 3 Royal (Dick) School of Veterinary Studies, University of Edinburgh, Edinburgh, UK

**Keywords:** Spinal muscular atrophy, Ribonucleoprotein, Translation, Cytoskeleton, Ubiquitin, Bioenergetic pathway

## Abstract

Ever since loss of survival motor neuron (SMN) protein was identified as the direct cause of the childhood inherited neurodegenerative disorder spinal muscular atrophy, significant efforts have been made to reveal the molecular functions of this ubiquitously expressed protein. Resulting research demonstrated that SMN plays important roles in multiple fundamental cellular homeostatic pathways, including a well-characterised role in the assembly of the spliceosome and biogenesis of ribonucleoproteins. More recent studies have shown that SMN is also involved in other housekeeping processes, including mRNA trafficking and local translation, cytoskeletal dynamics, endocytosis and autophagy. Moreover, SMN has been shown to influence mitochondria and bioenergetic pathways as well as regulate function of the ubiquitin–proteasome system. In this review, we summarise these diverse functions of SMN, confirming its key role in maintenance of the homeostatic environment of the cell.

## Introduction

The survival motor neuron (SMN) protein was first highlighted as a protein of interest when mutations in its coding gene, *SMN1,* were linked to the neuromuscular disease spinal muscular atrophy (SMA) [1], a leading genetic cause of infant mortality. SMA presents in a range of severities with the most severe form, Type 1, being fatal within the first 2 years of life. Patients show degeneration of α-motor neurons in the lower spinal cord leading to progressive muscle weakness. The clear importance of SMN protein to the motor system, alongside findings that knockout of *Smn* in mice was embryonically lethal [2], led to it being named “survival motor neuron”, despite subsequent research showing that it is a ubiquitously expressed protein, required by all cells and tissue types, not just neurons [3]. Over the past three decades, significant research efforts have sought to better understand the mechanisms through which SMN acts [3]. Most of the resulting knowledge has been generated from animal models of SMA, where reduced expression of SMN reveals its role in several important intracellular processes, which we will discuss in this review.

The full-length—294 amino acids, 38 kDa—human isoform of SMN (also known as FL-SMN, referred to as SMN hereafter) is mainly transcribed from the telomeric *SMN1* gene, located on chromosome 5q13. *SMN1* contains nine exons, 1, 2a, 2b, 3, 4, 5, 6, 7 and 8, with exon 8 remaining untranslated (Table 1). An inverted duplication in the region of *SMN1* resulted in a second centromeric copy of the gene, termed *SMN2*, an evolutionarily recent event unique to *Homo sapiens* [4]. *SMN2* differs from *SMN1* at 5 bases, and a C-to-T transition in exon 7 of *SMN2* favours skipping of exon 7 during splicing, resulting in the majority of *SMN2* products being a truncated isoform referred as SMNΔ7 [1, 5]. However, limited amounts of SMN can still be produced from the *SMN2* gene and it is known that the copy number of *SMN2* is inversely correlated with SMA disease severity [6]. Patients with homozygous null mutations of *SMN1* carrying four or more copies of *SMN2* show a less severe phenotype, later age onset, and can have a normal lifespan [7]. SMNΔ7 is highly unstable and quickly subjected to the ubiquitin–proteasome pathway for degradation [8, 9]. Phylogenetic studies further highlighted the importance of SMN, since it is highly conserved throughout evolution and there are even multiple copies of *SMN1* in the chimpanzee genome [4]. Other SMN isoforms have been found in various tissues (Table 1). An “SMN6B” protein can be translated from both the *SMN1* and *SMN2* genes by the inclusion of an intronic Alu sequence as an alternative exon following exon 6 [10]. SMN6B is present both in the nucleus and cytosol, and is twofold more stable than SMNΔ7 but twofold less stable than full-length SMN. SMN6B is able to interact with Gemin2, although its physiological function is not fully understood [10]. mRNA transcripts of another isoform, SMNΔ5 (with the exclusion of exon 5), can be found in muscle and the central nervous system (CNS) although, again, whether it has a physiological function is not clear [11]. An axonal-SMN (a-SMN) has also been proposed, being produced from intron 3 retention during splicing. Due to an in-frame stop codon in intron 3, a-SMN mRNA is much shorter and encodes a protein of 19 kDa. a-SMN is reported to be localised to the axon, and its expression is enhanced in the spinal cord and the brain during development and declines in the adulthood, with a hypothesised role in axonogenesis [12].

**Table 1:** Main isoforms of SMN, their composition, expression and localisation

SMN isoform	Splicing	Protein isoform	Expression	Localisation	References
Full-length SMN (FL-SMN)	Exons 1, 2a, 2b, 3, 4, 5, 6, 7, 8	Functional SMN protein	High expression during development, decreasing into the adult CNS	Nuclear gems and cytosolic, including axons, dendrites and synapses	[102, 205]
SMNΔ7	Exons 1, 2a, 2b, 3, 4, 5, 6, 8	Degradation signal introduced by the change in C-terminal	High expression during development, decreasing into the adult CNS	Nuclear accumulation	[104, 206]
Axonal-SMN (a-SMN)	Inclusion of intron 3	Truncated protein due to premature stop codon on the boundary of exon 3/intron 3	Expressed during development, not detected in the adult CNS	Motor neuron axons	[12]
SMN6B	Inclusion of an Alu element forming exon 6B	Truncated protein due to premature stop codon after exon 6B	Unknown	Nuclear and cytosolic	[10]
SMNΔ5	Exclusion of exon 5	Unknown	Expressed in the mature CNS	Unknown	[11]

Other splicing isoforms of SMN have also been discovered in cell cultures, although their role in vivo is yet to be determined. These include isoforms excluding exons 3, 4 and 5 or multiple exons both in stressed and normal conditions [207, 208]. Skipping of any internal exons of SMN maintains the reading frame

The SMN protein contains several functional motifs including, moving from N-terminus to C-terminus, a basic/lysine-rich domain, a Tudor domain, a proline-rich domain and a YG box. The basic/lysine-rich region is encoded by exon 2 and has been found to interact with Gemin2 and RNA in vitro and in vivo [13–16]. The Tudor domain is a highly conserved motif with a function in protein–protein interactions [17, 18]. The SMN Tudor domain binds to the C-terminal arginine- and glycine-rich tails of Sm proteins which contain symmetrical dimethylated arginine residues, thereby facilitating the assembly of spliceosomes as discussed later [18–24]. Mutations in this domain, which disrupt the interaction of SMN and Smith core (Sm) proteins, are often found in SMA patients [18, 25–27].

The Tudor domain of SMN is also responsible for an interaction with coilin, a marker of Cajal bodies (CBs) [28]. Liu and Dreyfuss originally described the localisation of SMN to nuclear bodies which they termed “gems” [29], and which are coilin negative as opposed to CBs. Gems are composed of SMN complex proteins, whereas CBs are more complex nuclear structures to which SMN also localises, and the presence of SMN in these nuclear bodies increases during neuronal differentiation [30]. CBs are enriched with small nuclear RNAs (snRNAs) and small nucleolar RNAs (snoRNAs), and are essential for the biogenesis of the small nuclear ribonucleoproteins (snRNP) complex [31]. Interestingly, motor neurons from a Type I SMA patient showed a reduced number of CBs and defects in recruitment of SMN and snRNP for spliceosomal maturation [32]. Tapia et al. [33] also demonstrated that SMN has a SUMO-interacting motif (SIM) in the Tudor domain, which is required for Sm protein binding and the assembly of CBs. Three polyproline stretches encoded by exons 4–6 are responsible for the binding of profilins, key proteins in the regulation of actin dynamics [34, 35]. A tyrosine/glycine-rich region in the C-terminus of SMN, termed the YG box, is found to facilitate oligomerisation of SMN by formation of the glycine-zipper structure [36]. Mutations found in the YG box count for nearly half of missense mutations in SMA patients and this motif was shown to be essential for survival in chick cells under conditions of SMN depletion [37, 38]. The YG box is required for SMN self-oligomerisation and proteins with mutations found in this motif, as seen in the Type I SMA patients, severely impair this association [39]. A recent study demonstrated that a phosphor degron within the YG box is exposed to SCF^Slmb^ ubiquitin E3 ligase when SMN is monomeric, implying that the YG box plays a role in SMN stability and indicating the importance of SMN oligomerisation [9]. Post-translational modifications to the SMN protein discern its localisation and function. As well as SUMOylation via the SIM regulating Sm protein binding, and ubiquitination of SMN (discussed later), the protein may also be acetylated, which promotes a cytoplasmic localisation and increases its half-life [40], or phosphorylated on certain serine/threonine residues to promote its localisation to CBs. Mutations of three tyrosine residues in the Tudor domain greatly affect its enrichment in CBs via disrupted interaction with coilin [41, 42].

Several model organisms have been utilised to study the SMN protein and its role in SMA (Table 2). Although the protein product of *SMN2* is truncated and unstable, its expression is crucial for survival once *SMN1* expression has been lost. As *SMN2* is specific to humans, most of the commonly used mouse models have undergone genetic manipulation to generate an endogenous Smn null mutation with concurrent overexpression of the human *SMN2* gene, a cDNA copy of *SMN2* lacking exon 7 or other variants of the *SMN* genes [43–45] (reviewed in [46]). Another widely used animal model is the zebrafish (*Danio rerio*). Having the advantages of well-characterised motor neurons and neuromuscular junctions, easy manipulation of gene overexpression or knockdown by injection of in vitro-transcribed RNA or antisense oligonucleotides, respectively, and a transparent body for imaging applications, zebrafish are becoming increasingly popular for translational SMA research projects [47–50]. Fruit flies (*Drosophila melanogaster*) have also been used to study SMN biology. They possess one copy of an *Smn* gene ortholog or *DmSMN* and can also be easily genetically manipulated. Each of the models referred to in this review are summarised in Table 2, with more comprehensive reviews of SMA models available elsewhere [51–54].

**Table 2:** Overview of animal models referred to in this review

Species	Endogenous *SMN* orthologue	Modelling strategy and/or genotype		References
*Caenorhabditis elegans*	*CeSMN*	Knockdown of expression through RNAi		[209]
*Drosophila melanogaster*	*DmSMN*	Point mutations or transposon insertions for knockout or knockdown studies		[210, 211]
*Danio rerio*	*Smn*	Knockdown of expression through antisense oligonucleotides		[47]
*Mus musculus*	*Smn*			
Smn knockout		*Smn* ^− */*−^	Smn null mutation by targeted insertion of β-galactosidase in Smn exon 2A	[2]
Taiwanese mice		*Smn* ^*H7/H7*^ *; SMN2Hung* ^*tg/*−^	Two copies of the *SMN2* transgene, *Smn* exon 7 is replaced with hypoxanthine phosphoribosyl transferase (HPRT) but transcripts without exon 7 are produced.	[43]
SMNΔ7		*Smn* ^*2A/2A*^ *; SMN2* ^*tg/tg*^ *; SMN∆7* ^*tg/tg*^	One copy of the *SMN2* transgene and one *SMNΔ7* transgene on Smn null background	[45]
Smn2B		*Smn* ^*2B/*−^	Mutation within the splicing enhancer of *Smn* exon 7 producing SMN2-like transcripts and reduced FL-SMN protein	[212]
Burghes severe model		*Smn*^−*/*−^; *SMN2*^*tg/tg*^	Smn null mutation by target replace of β-galactosidase in Smn exon 2A; with one copy of *SMN2*	[44]

For a comprehensive review of animal models of SMA, see Edens et al. [51]

The selective cell death of motor neurons is a key feature of the disease, but the reasons behind this selectivity are still poorly understood. A recent study demonstrated that there were heterogeneous and surprisingly diverse expression levels of SMN in SMA-patient-derived iPSC motor neurons. Moreover, motor neurons with lower levels of SMN protein were more susceptible to cell death from toxic compounds, whilst overexpression of SMN in motor neurons was protective [55]. SMN, therefore, clearly plays a major role in SMA pathology and the specific vulnerability to motor neurons in this disease. To understand why SMN is so vital for healthy cell maintenance, we must understand its role under normal, as well as disease, conditions. In this review, we describe the role of the SMN protein in regulating protein homeostasis. Protein homeostasis within cells can be regulated by two major pathways, production and clearance, which reach a dynamic balance to support and maintain physiological status. Production pathways incorporate protein translation, folding, modification and assembly, while protein clearance pathways are centred around protein disassembly and degradation. We discuss known functions of the SMN protein, starting with its first-described role in RNA splicing and spliceosomal assembly, followed by more recently discovered functions in regulating mitochondrial homeostasis, endocytosis and the cytoskeleton, ubiquitination and autophagy, and RNA transportation, thus giving a broad picture of the many ways in which SMN plays a key role in regulating protein homeostasis.

## SMN and ribonucleoprotein assembly

Although it is now clear that the SMN protein contributes to numerous cellular processes and pathways, the first identified and most studied function of SMN is its role in snRNP assembly. The spliceosome is a complex machine, which catalyses the removal of introns from pre-mRNA transcripts (see [56] for a detailed review). The biogenesis of an snRNP starts in the nucleus by the transcription of uridine-rich snRNAs (U1, U2, U4, U5, U6, U11, U12, U4atac, U6atac), which are then exported to the cytoplasm. Each snRNA is then bound to accompanying proteins and—with the exception of U6 and U6atac—to a set of seven Sm proteins. Whilst Sm proteins can spontaneously associate in vitro with almost any single short-stranded uridine-rich RNA forming a thermodynamically stable heptamer [57], this process lacks specificity regarding RNA substrates. The role of the SMN protein, tightly associated with eight other proteins (Gemin 2–8 and Unrip) in a large macromolecular SMN complex, is to chaperone the biogenesis of snRNPs from snRNAs and Sm proteins in the cytoplasm, and subsequent snRNP trafficking to the nucleus [14, 29, 58–66]. First, the SMN complex enforces specificity during the snRNPs’ assembly with the direct and specific binding of Gemin5 to the cytoplasmic snRNAs [67–71]. The Sm core is then loaded onto the snRNA in an ATP-dependent process with Gemin2 playing a key architectural role in this assembly. Finally, the snRNA undergoes hypermethylation of its m7G-cap by TGS1 (trimethylguanosine synthetase 1), leading to the formation of a trimethylguanosine (TMG) cap and trimming of its 3′ end before it can be imported back into the nucleus. The TMG cap and Sm core operate as nuclear localisation signals [72, 73]. Importation to the nucleus also necessitates the binding of the nuclear import complex (snurportin and nuclear import receptor importin-β) to the TMG cap [72, 74–77]. In that process, SMN has been shown to have a direct interaction with importin-β facilitated by WRAP 53 [78, 79]. WRAP 53 also plays a fundamental role in the trafficking of SMN towards CBs by facilitating the interaction between SMN and coilin [80]. The snRNA then dissociates from the SMN complex and undergoes its final maturation steps within the CB. Studies of splicing activity in cells from SMA patients or mouse models confirm the fundamental role of SMN in snRNP assembly with a correlation between the reduction in snRNPs levels and disease severity [81–83]. A recent study has also identified an alternative assembly pathway, whereby the U1-specific RNA-binding protein (RBP) U1-70K can directly interact with the SMN-Gemin2 complex, independently of Gemin5. This U1-specific Sm core-assembly pathway not only contributes to U1 overabundance, but it was also proposed that SMN-Gemin2 could play a role as a hub, where various RBPs and their RNA cargos congregate, hence promoting ribonucleoprotein exchange [84].

The SMN complex is also involved in the biogenesis of U7 snRNPs, a specific subgroup of snRNPs which are involved in processing the 3′ stem loop of histone mRNAs by endonucleolytic cleavage of the pre-mRNA sequence which immediately follows the hairpin [85]. The assembly of U7 snRNPs is overall analogous to that of the spliceosome snRNPs, with the exception of the slightly degenerate Sm-binding site of the U7 snRNA and the replacement of two of the Sm proteins in the Sm core (SmD1 and SmD2) by two U7-specific Sm-like proteins (Lsm10 and Lsm11). Similar to spliceosome assembly, the SMN complex is a specificity chaperone that is necessary to precisely recognise and combine U7 snRNA with the Sm heptamers containing Lsm10 and Lsm11, without which the U7 snRNPs cannot function in histone RNA processing [86–88].

Although less extensively studied, the SMN complex is suspected to be involved in the assembly and metabolism of other ribonucleotide complexes, including small nucleolar ribonucleoproteins (snoRNPs), associated with the post-transcriptional processing and modification of ribosomal RNA in the nucleolus (methylation and pseudouridylation) [89]. Indeed, SMN has been shown to directly interact with fibrillarin and GAR1, two markers of snoRNPs, and expression of a dominant-negative mutant of SMN results in abnormal accumulation of snoRNPs in large aggregates outside of the nucleolus [90]. Furthermore, in SMA-patient-derived cells, a decreased localisation in CBs of the snoRNP chaperone Nopp140 was observed, which correlated with disease severity [91]. In addition, SMN may be involved with telomerase, a large RNP complex that adds repeat sequences at the chromosomal ends. It comprises a telomerase reverse transcriptase (TERT), the telomerase RNA and other associated proteins (for a review on telomerase RNA, see [92]). The telomerase RNP belongs to the H/ACA snoRNP class and it is suspected that SMN plays a role in telomerase biogenesis, ensuring specificity of assembly and correct trafficking [93, 94].

As snRNP assembly and splicing occurs in all cells, including neurons, why do low levels of SMN in SMA particularly affect motor neurons [95]? This remains a major question challenging the SMN and SMA research field. As previously noted, the reduction in snRNP biogenesis correlates with the degree of clinical severity in SMA [81]. However, SMN deficiency seems to preferentially disrupt the formation of minor snRNPs, such as those responsible for the removal of U12-containing intron genes [81, 83, 96]. Amongst these, the gene coding for a transmembrane protein, stasimon, has been identified as being aberrantly spliced in a Drosophila model of SMA [97]. Upregulation of stasimon rescued deficient neuromuscular junction (NMJ) transmission in SMN-deficient Drosophila and improved neuronal development in SMN-deficient zebrafish [97]. Other genes, not all containing U12-introns, such as chondrolectin, agrin and neurexin2 have also been identified as being abnormally spliced and could, therefore, play a role in the pathophysiology of the disease [98–100]. It could consequently be the case that defects in splicing have a larger effect on a specific subset of neuronal genes, thereby rendering motor neurons particularly vulnerable. However, despite this evidence for mis-splicing in the SMA disease pathway, other studies have suggested that wide-spread splicing defects mainly occur during the late stage of the disease [101], supporting the theory that alternative roles of SMN may play an equally important part in cell function.

## SMN and trafficking

The first indications that SMN played a role aside from its canonical functions in the spliceosome came when electron microscopy revealed localisation of SMN in the dendrites and axons of motor neurons in the developing rat spinal cord [102]. It has been suggested that there is a progressive shift in SMN protein localisation from mainly nuclear during development to a more cytoplasmic and axonal localisation in the mature neuron [103]. SMN was also found to be present at the growth cones of cultured motor neurons, and live cell imaging showed puncta positive for SMN being actively transported bi-directionally along axons [104]. SMN co-localises with some elements of the SMN complex in the axon, such as Gemin2, but Sm proteins show very low abundance in distal neurites, and most axonally located SMN granules lack Sm proteins [105]. The neuron-specific protein neurochondrin is required for the correct localisation of SMN in the cytoplasm, and neurochondrin was found not to co-localise with snRNPs, further indicating that SMN is involved in activities other than splicing [106].

Recent studies have identified that SMN can bind to the α-COP subunit of the COPI vesicle [107]. The COPI system, a Golgi-derived vesicular transport system, is involved in intracellular trafficking in neurites, necessary for the maturation of neuronal cell processes [108]. Knocking down α-COP was found to disrupt SMN localisation within growth cones, resulting in its accumulation within the trans-golgi network [109]. Depletion of α-COP reduced neurite formation in NSC-34 cells and primary cortical neurons, with shortening of both map2-positive dendrites and tau-positive axons [110], and both α-COP and SMN are required for correct neurite formation [111]. This indicates a role for SMN in trafficking for the purposes of neuronal outgrowth and formation of the axonal and synaptic cytoskeleton (see below).

In keeping with this potential role for SMN, Rossoll and colleagues discovered an interaction between SMN and the RBP hnRNP-R [112]. SMN and hnRNP-R were found to co-localise in the cytoplasm of primary cultured motor neurons, and in motor neurons cultures from Smn^−/−^ mice, there was a large reduction in β-actin mRNA localisation in axons and growth cones. Primary motor neurons cultured from Taiwanese SMA mice showed growth cones with a threefold reduction in size compared to healthy controls, as well as reduced staining for β-actin mRNA with no overall change in protein expression [112]. Since these initial findings, fluorescence in situ hybridisation experiments against the polyA tails of mRNA revealed a more than 50% reduction in localisation of mRNA transcripts along the axon of primary motor neurons following SMN knockdown [113]. In addition, further co-localisation studies have shown SMN to associate with a number of RBPs via its Tudor domain, including KSRP [114], FMRP [115], HuD [113, 116], FUS [117] and IMP1 [118]. The association between SMN and other RBPs has linked it to another motor neuron disease, amyotrophic lateral sclerosis (ALS). RBPs associated with ALS, FUS and TDP-43 have been shown to co-localise in nuclear gems with SMN and mutations in either of their genes in ALS patient fibroblasts show reduced gem formation leading to abnormal accumulation of snRNAs in the nucleus [119, 120]. This highlights an interesting mechanistic link between ALS and SMA.

SMN acts as a molecular chaperone for the binding of RBPs to mRNA transcripts as well the RBPs’ binding to the cytoskeleton and subsequent localisation, as evidenced by disruption of these processes in SMN deficiency [121]. Both IMP1 and HuD have been shown to influence the localisation and translation of β-actin and GAP-43 mRNA transcripts, which are in turn both necessary for correct axonal growth [113, 118, 122]. Indeed, SMN knockdown leads to a reduction of HuD in the axonal compartment [113], while knockdown of HuD in zebrafish leads to a similar phenotype to SMN knockdown of shorter axons [122]. Further experiments using FLAG-tagged SMN in NSC-34 cells [123] identified SMN as a binding partner for several species of non-coding RNAs, including snRNAs, snoRNAs and ribosomal RNAs, which were expected due to SMN’s known role in RNA processing, as well as miRNAs and tRNAs. The majority of RNAs identified were mRNA, with many being part of ribosomal and/or metabolic pathways. When compared to mRNAs known to localise to neuronal axons, the study’s authors identified 75 axonally localised SMN-associated mRNAs, including RNA transcripts of several ribosomal proteins and Ubb, the transcript of the protein ubiquitin [123]. However, it should be noted that this group of mRNAs is unlikely to be comprehensive, as it does not include the known RNA transcripts regulated by SMN including β-actin, GAP43, tau and neuritin [112, 116, 124].

## SMN and translation

While SMN plays an integral role in the transport of RNA transcripts along axons and dendrites, it also appears to be involved in the local translation of proteins. The transportation of mRNA transcripts along the axon allows for rapid protein turnover in distal regions of the neuron in response to, for example, activity [125]. Dysregulation of local translation has been associated with several other neurodegenerative disorders, including Alzheimer’s disease and amyotrophic lateral sclerosis (reviewed in [126]).

Recent evidence has pointed to a role for SMN in the local translation of mRNA transcripts, as well as their localisation. Early on, it was reported that loss of SMN changed the expression of plastin-3 in a zebrafish model of SMA [127]. Although, at the time of this discovery, the mechanisms behind changes in protein expression were not clear, more recent studies suggest that SMN affects the local translatome through several mechanisms. Translation within the axon was found to be dysregulated in primary neurons derived from SMN^−/−^ mice in vitro, with no corresponding change in somatic translation, and axonal defects could be rescued by overexpression of the RNA binding proteins HuD and IMP1 [128], suggesting a link between the role of SMN in mRNA trafficking and translation. Furthermore, ultrafractionation of cell extracts from a motor neuron-like cell line revealed an association of the SMN protein with polyribosomes, whilst treatment with RNase displaced RBPs associated with the polyribosomes such as SMN, but also other known binding proteins such as FMRP [129]. When SMN was introduced to an in vitro translation system, there was a dose-dependent reduction in translation efficiency, with no change in translation when incubated with the SMNΔ7 fragment [129].

Alongside its direct interaction with ribosomes, suggesting a possible direct role in translational control, SMN may also influence protein translation through micro-RNAs. MicroRNAs miR-183, miR-96 and miR-182 are transcribed in a cluster and are associated with increased cell proliferation via the mTOR pathway [130]. Primary motor neurons with a 50% knockdown of SMN protein showed increased expression of miR-183 in neurites whereas there was no change in expression in the cell body, along with downregulation of proteins in the mTOR pathway [131]. It is possible that SMN regulates the mTOR pathway and, therefore, protein translation through miR-183, since motor neurons in the Taiwanese mouse model and SMA-patient-derived fibroblasts both showed reduced levels of de novo protein synthesis, and a knockdown of miR-183 in Taiwanese mice produced a mild rescue of the phenotype with improved survival and increased body weight [131]. Another indication that SMN interacts with the mTOR pathway came from studies examining the effect of manipulating the PTEN pathway on primary motor neurons of SMNΔ7 mice. PTEN is a negative regulator of the mTOR pathway, and in SMN-depleted primary motor neurons where axonal growth was defective, decrease of PTEN/activation of mTOR rescued the SMA phenotype [132].

Most of the work detailed above was performed in vitro, where primary cultures allow analysis of changes in axonal growth and the ability to isolate axonal compartments relatively easily. However, in vivo evidence is important to determine the role of SMN in these mechanisms. A recent study examined translational pathways in vivo and found SMN associating with ribosomes in control tissue, as well as a shift in residual SMN levels to non-ribosomal fractions and an overall reduction in the number of ribosomes associated with polysomes in Taiwanese SMA mice [133]. This study also compared the whole transcriptome to the translatome using next-generation sequencing, and confirmed significant deficiencies in translation following reduced SMN expression in vivo [133], including results that point to defects in the biogenesis of ribosomes, suggesting a possible explanation for translational defects that occur upon SMN depletion.

Taken together, the studies detailed above strongly suggest that SMN plays an important role in regulating protein translation through several mechanisms. First, through subcellular localisation of mRNAs along the axon; second, through association with ribosomes themselves regulating the availability of ribosomal units for local translation and finally, through regulation of the mTOR pathway. In this way, SMN is well positioned to play a role in the developmental polarisation of motor neurons, as well as control their growth, maturation and proper function. This crucial role, alongside the expansive physical size of motor neurons, may partly explain why motor neurons are particularly susceptible to loss of SMN, as opposed to other more ubiquitous roles that the protein plays in the cell.

## SMN and the cytoskeleton

The cytoskeleton—incorporating key components such as microtubules, neurofilaments and actin protein—plays a fundamental role in regulating neuronal architecture and function. It is crucial for signalling and trafficking of various molecules, but also for the formation of growth cones during neuronal development. Therefore, it is perhaps not surprising that defects in the cytoskeleton have been linked to several neurodegenerative disorders, including SMA (for reviews on the neuronal cytoskeleton see [134, 135]).

The observation that SMN localised to neurites and growth cones [102, 105, 136–138] and that SMN modulated the localisation of β-actin within growth cones [112] provided the first hints of a possible role for SMN in regulating cytoskeletal dynamics. At the same time, knocking down SMN in zebrafish was found to result in motor axon pathfinding deficits [47], whilst SMN-deficient cell cultures showed neurite extension defects [104, 112, 139, 140]. The fact that selective overexpression of the SMN C-terminal domain could rescue these neurite deficits in SMN-deficient PC12 cells argued in favour of a role of SMN in microfilament metabolism independent of snRNP biogenesis, as the Tudor domain where Sm proteins binds was not present in this C-terminal construct [139].

**Fig. 1:**
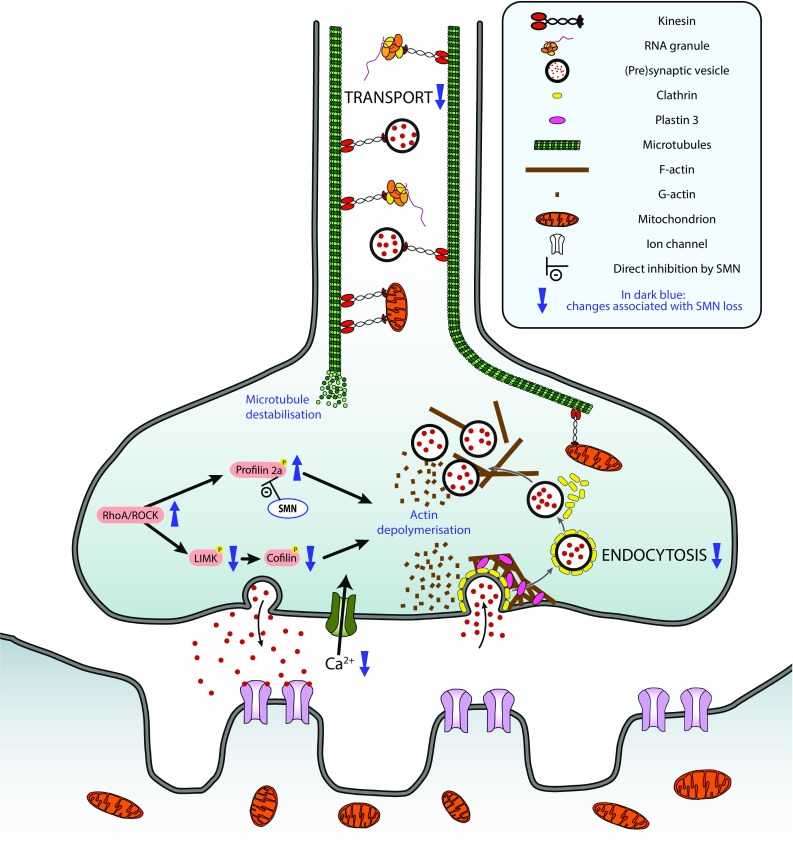
Schematic overview of the alterations in cytoskeletal dynamics and endocytosis observed following SMN deficiency. The diagram highlights these changes at the level of the motor neuron and neuromuscular junction. All changes associated with SMN loss are represented in dark blue. SMN deficiency results in a decrease in cellular transport (e.g. synaptic vesicle, RNA granules and mitochondria) and endocytosis. In the absence of SMN, not only a destabilisation of the microtubules is observed, but also a depolymerisation of the actin cytoskeleton, which has been linked to the activation of the RhoA/ROCK pathway

Growth cone formation and neurite extension are mediated by actin dynamics, and SMN has been found to colocalize with profilin 2a during neurite cell extension [141] and in nuclear gems [34]. Profilin 2a is an actin-binding protein primarily expressed in the nervous system where it is involved in the regulation of actin turnover by promoting actin polymerisation. Profilin 2a binds to a stretch of proline residue within the SMN protein [35], and this interaction with SMN modulates the activity of profilin. Profilin 2a is also a known downstream target of the rho-kinase (ROCK) pathway, a key regulator of actin dynamics (reviewed in [142]). Knocking down SMN in PC12 cells resulted in an upregulation of profilin 2a, which, combined with its increased availability due to decreased interaction with SMN, lead to an upregulation of the ROCK pathway with subsequent inhibition of neuronal outgrowth [143]. ROCK pathway inhibition in an intermediate SMA mouse model (Smn2B) also resulted in increased life span and amelioration of muscle pathology [144] (see Fig. 1 for a summary of the role of SMN in cytoskeleton dynamics). Moreover, it was recently suggested that SMN loss resulted in the dysregulation of the actin cytoskeleton by interfering with PlexinD1. PlexinD1 is a receptor for class 3 semaphorins and acts as a signalling factor to guide axonal growth. In the Taiwanese mouse model and in iPSC-derived motor neurons from SMA patients, PlexinD1 was shown to be cleaved by metalloproteases, resulting in its functional change from being an attractant to a repellent signalling factor, thereby contributing to growth cone collapse [145]. In the same study, cleaved PlexinD1 was found to be enriched in actin rods, a pathological structure of elongated actin aggregates also found in some age-related neurodegenerative diseases but not in control cells.

Interestingly, by studying discordant families where siblings of SMA patients were asymptomatic despite carrying the same *SMN1* and *SMN2* alleles as their affected siblings, Oprea et al. [146] were able to identify the first protective genetic modifier of SMA: plastin 3 (PLS3). PLS3 is involved in axonogenesis by bundling F-actin and stabilising growth cones. Its overexpression was able to rescue the axon outgrowth defects in SMN-deficient zebrafish and increase the life span of the intermediate Smn2B model [146, 147]. Other studies have suggested additional roles for SMN in regulating microtubule formation, required for transporting mRNAs, proteins and organelles to or from the nucleus to distal regions of the neuron (reviewed in [148]). Stathmin, a protein known to promote microtubule depolymerisation [149] was found to be upregulated in the spinal cord in Taiwanese mice and also in SMN-depleted NSC-34 cells leading to defects in the structure of axons and reduced mitochondrial transport along the axons [150]. In SMN-deficient cells, microtubules failed to re-polymerise following treatment with the microtubule-depolymerisation agent nocodazole, an effect which could be rescued by knocking down stathmin [150]. However, the detailed mechanisms linking SMN to these pathways, and whether or not they are indeed separate from SMN’s involvement in the mRNA trafficking of components of the cytoskeleton such as GAP-43 and β-actin, remains to be determined.

## SMN and endocytosis

Endocytosis is a basic cellular process, essential for neuronal signalling, axonal and dendritic growth (reviewed in [151]). It plays a particularly important role at synapses (including at neuromuscular junction synapses formed by motor neurons), facilitating synaptic vesicle recycling necessary for repeated rounds of neurotransmitter release. A bioinformatics analysis carried out on two different species (*Caenorhabditis elegans* and *D. melanogaster*) identified the endocytic pathway, along with mRNA regulation, as potential modifiers of SMN loss [152], with numerous individual genes being highlighted. In another study, SMN depletion resulted in a marked impairment of endocytic function in multiple tissues of *C. elegans* [153]. The neuromuscular junction was particularly affected, with structural and functional changes being reported. A reduction in the number of pre-synaptic docked vesicles was observed, accompanied by unusually large cisternae suggestive of arrested endocytic vesicle maturation [153]. This was associated with a decreased activity of, and disruption to, the NMJ (a key feature of SMA [154–156]): synaptic transmission was reduced, likely secondary to an impairment in synaptic vesicle recycling. In this model, endosomal defects were noted not only at the level of the NMJ, but also in non-neural tissue as endocytic activity in coelomocyte cells was lower. The importance of SMN for NMJ homeostasis was further demonstrated in the Taiwanese model of SMA, where pre-synaptic uptake of FM1-43 dye by endocytosis was significantly reduced upon electrical stimulation. Interestingly, this disturbance was restored by PLS3 overexpression. The fact that PLS3 overexpression could improve the endocytic defect was perhaps not surprising, however, as the actin cytoskeleton is required for this process [157] and yeast cells lacking Sac6p, the PLS3 ortholog, are defective for the internalisation step of endocytosis [158]. Moreover, another F-actin binding and bundling protein, coronin 1C (CORO1C), has been shown to interact with PLS3 and its overexpression rescued endocytosis in SMN-deficient cells and improved the axonal phenotype in Smn-deficient zebrafish [159]. The importance of SMN for endocytic processes has also been confirmed in SMA-patient-derived cells, which proved resistant to infection by a clathrin endocytosis-dependent virus [153] (see Fig. 1 for a summary of the role of SMN in endocytosis).

Using the same approach that led to the discovery of PLS3 as a modifier of SMA, a second modifier, neuronal calcium sense protein neurocalcin delta (NCALD) was recently reported [160]. Contrary to PLS3 which acts as a positive regulator of endocytosis, NCALD is a negative regulator of endocytosis and axonal growth. Knockdown of NCALD restored neurite outgrowth in SMN-deficient cells and improved axonal growth and NMJ function in a zebrafish model of SMA. An enhanced neuromuscular function in *C. elegans* and murine models of SMA was also observed following NCALD depletion [160]. In the absence of calcium or at low calcium levels, NCALD, which localises to growth cones and pre-synaptic sites at the NMJ, interacts with clathrin, which mediates the endocytosis needed for fast recycling at axon terminals. Low SMN levels have been shown to lead to a reduction of voltage-activated Ca^2+^ influx [98, 160], and it is possibly through this mechanism that endocytosis and vesicle recycling was impaired. It was postulated that, in normal motor neurons, the high local Ca^2+^ concentration observed following neurotransmitter release led to the dissociation of NCALD from clathrin, therefore “freeing” clathrin to perform its endocytic function. In SMA, due to low Ca^2+^ concentrations, dissociation did not occur and the clathrin was, therefore, not available for coating of the vesicles. Moreover, disturbed calcium homeostasis would also be predicted to affect the function of actin-bundling proteins PLS3 and CORO1C, giving further strength to the hypothesis that low calcium levels secondary to SMN deficiency play an important role in endocytosis impairment [158].

## SMN and autophagy

Autophagy is a highly conserved catabolic process utilised by cells to break down unwanted macromolecules such as aggregated proteins or cellular organelles (reviewed in [161]). Autophagy involves a double-membrane bound structure engulfing target proteins and organelles to form an autophagosome. The autophagosome later fuses with lysosomes to become an autolysosome, in which the proteins and organelles are degraded (reviewed in [162]). Autophagy is a finely balanced mechanism: a decrease in expression of autophagy-related genes may lead to the accumulation of unwanted proteins whereas over-active autophagy leads to increased numbers of autophagosomes, possibly leading to cell death [163, 164]. Both of these outcomes have been described in various models of SMN depletion, indicating a role for SMN in the regulation of autophagy.

It is debatable whether an increase in amount of autophagosomes is protective or deleterious to the cell. Through measuring expression of LC3-II, a marker of autophagosomes, it has been shown that autophagosome number is increased in primary motor neurons following lentiviral SMN knockdown [165] and in spinal cords of the Taiwanese mouse model [166] and the SMNΔ7 mouse model [167]. Another way of measuring autophagic activity is through autophagic flux indicated by the level of p62/SQSTM1 protein [168–170]. Again, the p62 protein level was found to be upregulated in the spinal cord of Burghes severe SMA mice compared to their control littermates [171], as well as in an NSC-34 cell line following lentiviral SMN knockdown, and in the spinal cord of Taiwanese SMA mice [166], indicating a reduction in autophagic flux. Inconsistent with data from the Taiwanese mouse model, autophagic flux did not appear to increase in the spinal cord of SMNΔ7 mice [166, 167]. Inhibition of lysosomal proteolysis with Bafilomycin A1 (BafA1) resulted in an accumulation of LC3-II in cultured motor neurons from the Burghes severe model, suggesting that SMN deficiency can activate autophagy [171].

Conversely, autophagy modulators can alter SMN protein levels. Treating cultured motor neurons isolated from wild-type mice with mTORC1 inhibitor rapamycin, which is believed to enhance the activity of autophagy [172, 173], showed increased SMN levels, whilst in BafA1-treated motor neurons SMN levels were decreased [171]. A recent study has indicated that SMN may be partially degraded through the autophagy pathway, since a knockdown of p62 in stem cell-derived motor neurons from SMNΔ7 mice increased SMN protein levels [174]. A role for SMN in autophagy is also supported by the finding that overexpression of the SMN-binding partner α-COP, normally involved in cytoskeletal growth [110], partially restored autophagic flux in SMN-depleted cells [166], although the mechanism involved remains unclear. Moreover, injection of the autophagy inhibitor 3-methyladenine (3-MA) into SMNΔ7 mice at P3 greatly reduced autophagic activity and protected motor neurons from degeneration, possibly via inhibition of the apoptotic pathway as shown by reduced expression of apoptotic markers [167]. On the other hand, rapamycin failed to influence the loss of motor neurons, but reduced survival significantly in SMNΔ7 mice [167]. These conflicting findings show that further work is still required to fully elucidate the interaction between SMN and autophagy pathways.

## SMN, mitochondrial homeostasis and bioenergetics pathways

SMN deficiency has been linked to changes in oxidative stress, mitochondrial dysfunction and impairment of bioenergetic pathways. Acsadi et al. [175] showed that knocking down SMN levels by ~ 66% in NSC-34 cells resulted in a marked reduction in ATP levels. This was associated with an increase in cytochrome c oxidase activity and mitochondrial membrane potential, resulting in increased free radical production. This increase in oxidative stress in SMN-deficient cells was further confirmed in spinal motor neurons derived from human embryonic stem cells (hESCs). Interestingly, mitochondrial superoxide production was only increased in the SMN-knockdown hESCs which were made to differentiate into spinal motor neurons, but not in the cells differentiated into forebrain neurons [176].

Further analysis of mitochondrial dysfunction was performed by the same group using two models of SMN-deficient cells, SMA Type 1 patient-specific-induced pluripotent stem cells (iPSCs) and SMN-knockdown hESCs, both differentiated into spinal motor neurons [177]. Impaired mitochondrial axonal transport and a reduction in axonal mitochondrial number and area were noted at early stages of cell culture. Partial rescue by the anti-oxidant N-acetylcysteine provides evidence to support the hypothesis that oxidative stress plays an important role in neuronal degeneration in SMN-deficient motor neurons. However, experiments on SMA patient iPSCs led to conflicting results as, in this model, no oxidative stress was detected [178]. These inconsistencies could be secondary to differences in the way the stem cells were differentiated and highlight the limitations of studying cell type-specific pathological processes in cell cultures. More recently, studies in SMNΔ7 and Taiwanese mouse models confirmed marked mitochondrial dysfunction in spinal motor neurons, with decreased basal and maximal mitochondrial respiration, impaired mitochondrial membrane potential, impaired mitochondrial mobility, increased oxidative stress level and increased fragmentation [179]. Interestingly, mitochondrial defects in SMA are not thought to be limited to motor neurons in vivo, as they have also been identified in SMA patient muscle associated with a downregulation of mitochondrial biogenesis regulatory factors [180].

Mitochondrial oxidative phosphorylation is a core part of bioenergetic pathways. Mitochondrial electron transport chain function relies on a supply of electrons from the carriers NADH and FADH_2_ through upstream reactions (mainly glycolysis and TCA cycle). Proteomics studies identified that bioenergetics pathways were affected by SMN deficiency, more specifically GAPDH, an enzyme of the glycolysis pathway, was downregulated in SMA models [181]. Interestingly, gene expression studies of affected and disease-resistant motor neuron pools in mice revealed that susceptible neurons had lower basal expression not only of specifically mitochondria-related genes but also of genes involved in more generic bioenergetic pathways. Specifically, the expression of PGK1, a key enzyme of the glycolytic pathway, was significantly elevated in motor neurons that are intrinsically resistant to low levels of SMN, with experimental elevation/activation of PGK1 sufficient to rescue motor axon defects and loss of neuromuscular function in a zebrafish model of SMA [182].

Taken together, these studies highlight that SMN deficiency leads to impairment in mitochondria and bioenergetics pathways. However, the precise mechanisms involved in these interactions remain unclear. Studies in various cell types have shown that SMN does not localise to mitochondria [175, 183]. Therefore, it has been postulated that the effects of SMN on mitochondrial function could be indirect, possibly by affecting preferentially the splicing, translation or mRNA transport of genes fundamental to mitochondrial homeostasis [175, 177]. As previously mentioned, cytoskeletal changes can also lead to decreased mitochondrial transport, particularly within long axons [150]. Therefore, further studies are required to better understand how SMN affects these energetic pathways, fundamental for cellular homeostasis.

## SMN and ubiquitin pathways

Another key mechanism required for protein homeostasis is the protein degradation pathway. There are two major routes of protein degradation in eukaryotes: the ubiquitin–proteasome system (UPS) and lysosomal proteolysis, or autophagy (see above). The mammalian ubiquitin pathway is initiated by activation of the E1 ubiquitin-activating enzyme UBA1, which then transfers ubiquitin onto one of around 40 E2 conjugating enzymes. E2 ligases control whether a substrate is mono- or polyubiquitinated [184]. E3 ligases (of which there are several hundred) collect the substrate protein and form a complex between it and the ubiquitinated E2 ligase, where the ubiquitin is transferred onto the protein substrate. Ubiquitination is a dynamic process, and proteins can be stripped of their ubiquitin by deubiquitinating enzymes.

SMN has been shown to be ubiquitinated and ultimately degraded via the ubiquitin–proteasome system, with a protein half-life of between 6 and 10 h depending on the cell line analysed [185, 186]. Inhibition of the proteasome in SMA-patient-derived fibroblasts increased the intracellular abundance of SMN, both in terms of the amount of SMN protein and the number of nuclear gems [187]. Monoubiquitination, as opposed to polyubiquitination, serves other functions in the cell instead of degradation including protein trafficking and intracellular localisation (reviewed in [188]) and SMN is known to be monoubiquitinated [186]. Indeed, preventing the monoubiquitination of SMN changed the localisation of the protein from the cytoplasm to the nucleus, and also prevented its co-localisation with Sm proteins [189]. Meanwhile, the SMNΔ7 fragment is polyubiquitinated and quickly degraded [186]. Pharmacological inhibition of ubiquitination of SMN, such as with the small molecule ML372, increased SMN protein levels and slowed disease progression of SMNΔ7 mice leading to longer survival, increased motor neuron size and less muscle atrophy [190]. When SMA-patient-derived fibroblasts were treated with salbutamol, the β_2_-adrenergic receptor agonist, there was also an increase in levels of SMN protein, possibly acting via activation of protein kinase A, thereby preventing SMN ubiquitination [191]. SMA patients treated with salbutamol also showed an increase in SMN levels in the blood [192].

**Fig. 2:**
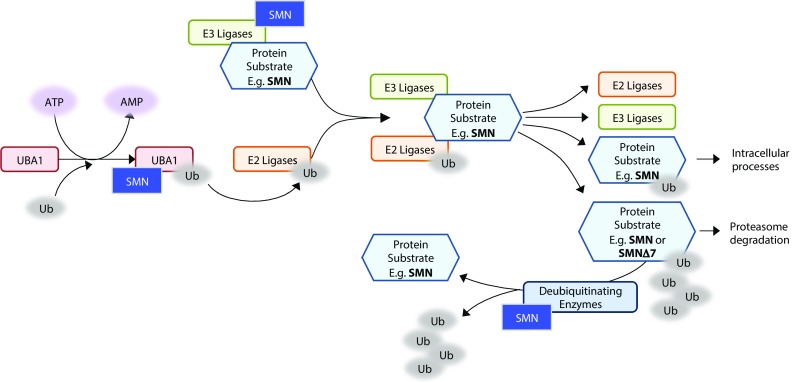
Diagrammatic representation of the ubiquitin pathway and the components, where SMN interacts. SMN is both ubiquitinated via the UPS pathway and an interacting protein influencing several steps of the process. SMN directly interacts with the UBA1 enzyme, which transfers ubiquitin to the E2 ligases. Ubiquitinated E2 ligases then form a complex with E3 ligases bound to protein substrates. SMN has been shown to interact with several E3 ligases, including Mindbomb 1, Itch and TRAF6. Ubiquitin is then transferred to the protein substrate and the complex dissociates. Monoubiquitinated substrates continue on to other intracellular processes, whereas polyubiquitinated substrates are targeted for proteasome degradation. SMN has also been shown to interact with deubiquitinating enzymes, which remove ubiquitin from protein substrates

Through proteomic analysis, SMN has been found to interact with several components of the ubiquitin pathway, including UBA1 and several E3 ligases, as summarised in Fig. 2 [9, 193]. Mutations in the *UBA1* gene cause the disease X-linked SMA [194], a rare condition with similar symptoms to classical SMA but with no mutations in the *SMN1* gene, suggesting a link between UBA1 and SMN which, when lost, leads to SMA-like phenotypes. Mutations in the Drosophila homologue of UBA1 cause motor defects, indicating that the motor system is particularly susceptible to the loss of UBA1 despite its ubiquitous expression [195]. Proteomic analysis of hippocampal synaptosomes from Burghes severe SMA mice showed decreased levels of UBA1 compared to controls, with decreased expression also reported in spinal cord and skeletal muscle [196]. The Taiwanese SMA mouse model similarly showed tissue-wide lower levels of UBA1, along with changes in splicing of the UBA1 transcript, which may account (at least in part) for the altered protein expression. Experimental suppression of UBA1 in wild-type zebrafish was sufficient to phenocopy SMA-like motor axon defects. Likewise, in the zebrafish SMA model UBA1 expression was reduced by 70%, whilst increasing UBA1 expression rescued the SMN-knockdown phenotype [197]. Finally, treating Taiwanese mice with an AAV9-UBA1 expression vector improved the survival, weight gain and motor performance of the mice as well as rescuing motor neuron cell number in the spinal cord and neuromuscular junction pathology [197].

As well as UBA1, SMN is known to interact with several other ubiquitin-associated enzymes (Fig. 2). Several E3 ubiquitin ligases have been shown to interact with SMN and so may be involved in its degradation through recruitment into the UPS. For example, Mindbomb 1 directly interacts with SMN [190]. Overexpression of Mindbomb 1 was shown to increase the amount of ubiquitinated SMN protein in cell culture, while a knockdown of Mindbomb 1 in the *C. elegans* model of SMA improves the SMN-deficient phenotype of defects in pharyngeal pumping [198]. Other E3 ligases known to interact with SMN include Itch [189], TRAF6 [199] and the Drosophila E3 ligase SCFslmb [9]. Monoubiquitination following interaction with Itch was shown to regulate SMN’s intracellular localisation [189]. TRAF6 activity is apparently inhibited by SMN, and so SMN binding may be involved in the activation of NF-κB signalling further downstream [199].

Ubiquitin carboxy-terminal hydrolase L1 (UCHL1) is a deubiquitinating enzyme specifically expressed in neuronal tissue, and its downregulation has been associated with Parkinson’s and Alzheimer’s diseases [200–202]. Following knockdown of UCHL1 in cell culture, there was a concordant increase in SMN expression [203]. Conversely, in Taiwanese mice, there was an increase in UCHL1 expression. However, inhibition of UCHL1 expression in Taiwanese mice failed to increase SMN levels and did not have an effect on survival or phenotype of the SMA model, with evidence suggesting that an increase in UCHL1 levels in the absence of SMN may be a compensatory response to restore levels of ubiquitination [204]. Usp9x, another deubiquitinating enzyme known to interact with SMN, also influences its ubiquitination levels, where a loss of Usp9x impairs SMN nuclear gem formation while overexpression leads to an increase in ubiquitinated SMN [186]. It, therefore, appears that SMN is regulated at several levels of the UPS, which may have an effect on cell-wide ubiquitination as well as regulation of the SMN protein itself.

## Concluding remarks and future perspectives

SMN, originally discovered due to its association with the neurodegenerative disorder spinal muscular atrophy, is in fact a ubiquitous protein with numerous roles within the cell. Although its first-identified and most-described function is in the biogenesis of ribonucleoproteins, it is now evident that SMN plays a more general housekeeping role. With this in mind, here we have discussed various areas of intracellular homeostasis in which SMN has been shown to interact: its well-known role as part of the ribonucleoprotein complex, but also other stages of RNA processing such as transport and local translation, important neuronal functions such as cytoskeletal dynamics and endocytosis, protein turnover processes of autophagy and ubiquitin–proteasome pathway and regulation of mitochondrial activity. Through tradition and necessity, the majority of current research into the function of SMN comes from SMA models of SMN deficiency. However, as this review has highlighted, SMN function is involved in so many aspects of normal intracellular activity that future SMN research should move beyond its association with disease to better understand its role in maintaining the homeostatic environment of the cell. Two major questions need answering in terms of the function of SMN. First, to what extent is SMN involved in the regulation of processes discussed in this review. While some areas have been researched extensively, such as ribonucleoprotein production, other areas of SMN involvement are a relatively new discovery, such as the association of SMN with mitochondrial function and ubiquitin degradation, and so further exploration is needed. Secondly, the particular vulnerability of motor neurons in SMA patients cannot be ignored. Although the idea that SMA is in fact a systemic disease, with defects seen across tissue types, is gaining acceptance in the research community, a better understanding of the multiplicity of SMN functions could serve to highlight areas of particular susceptibility in motor neurons which lead to their cell death in SMA. As SMN is at the cornerstone of so many molecular pathways, fundamental research into these cellular homeostasis processes is crucial to the better understanding of cellular biology.
